# Report of a Case of Anthrax

**Published:** 1901-09

**Authors:** Amand Ravold

**Affiliations:** St. Louis, Mo.


### REPORT OF A CASE OF ANTHRAX.*

BY AMAND RAVOLD, M.D.,

ST. LOUIS, MO.

The patient, Mrs. Z., age thirty-eight years, white, married, mother of two children, has suffered for some years with chronic nephritis; is fairly well nourished but anemic. September 29th, while scrubbing a slate drainage board attached to one side of her kitchen sink, ran a small splinter of slate into the outer surface of the first phalanx of the little finger of her right hand; the splinter was a small one and broke off even with the surface of the skin; she dug out the splinter with a pin which she heated in a gas flame before using; the splinter was very friable and she experienced a good deal of trouble in removing it, but afterwards continued at work. That night the wound pained enough to interfere somewhat with sleep, and by noon the next day, twenty-four hours after the injury, the pain, now of a throbbing character, was so severe that she came to me for relief.

* Read before the Medical Society of City Hospital Alumni, April 4, 1901.

The site of the splinter could be made out distinctly by a dark line, about seven millimeters in length, beneath the skin ; it seemed to me a very trivial wound to be the source of so much suffering, nevertheless I opened it up with a sharp bistoury. The small amount of debris along the line made by the splinter was carefully cleaned out, and the wound washed out with a solution made up of five per cent. carbolic acid, to which is added corrosive sublimate sufficient to make $\frac{1}{1000}$, and hydrochloric acid five per cent. This is a strong and penetrating disinfectant. The solution was rubbed into the wound and kept in it for about five minutes; the finger was then washed in sterile water, wrapped in corrosive sublimate gauze covered with rubber tissue and a bandage. Patient left with the pain very much relieved.

October 3d, she came to my office and said that up until that morning she had been free from pain in the wound, but that now it was of a throbbing character and as severe as when I first saw her. She certainly had the appearance of one suffering severe pain. Inspection shows a slightly inflamed wound with a small drop of pus in its depth. This was cleaned out, the wound again disinfected with the carbolic acid and corrosive sublimate solution, dressed as before and the patient left practically free from pain, with instructions to wash the wound every morning with a $\frac{1}{2000}$ solution of corrosive sublimate, and to dress it with gauze and rubber tissue, of which I gave her enough to last several days.

October 8th, five days later, she again came to my office complaining of a throbbing pain in the wound which had begun in the night. I found the finger much swollen and indurated. The wound was nearly healed, with no pus in it, but a vesicle had formed about it and extended across the upper surface of the finger, between the nail and joint, then down along the inner surface of the finger as far as the second joint. I split open the vesicle, washed it out with a $\frac{1}{2000}$ solution of corrosive sublimate, and dressed it with powdered boracic acid, gauze, rubber tissue and bandage. I requested her to return the next day, for I feared that I had poisoned her with the carbolic acid and corrosive sublimate solution, and had another case to add to the long list of dermal poisoning with carbolic acid.

I did not hear from her until early on the morning of October 11th, when I was called to the patient's home, and found her in bed recovering from an hysterical attack brought on, she said, the night before by the throbbing pain in the little finger, and in which she had been attended by Drs. J. M. Grant and S. Klein. She now complained of intense pain in the wound; I removed the dressing which she had about the finger and hand, and found the finger, hand and forearm much swollen, with the glands in the axilla tender but not perceptibly enlarged; a hemorrhagic vesicle had formed in the area of the first vesicle and the skin about the edges of the vesicle was black and necrotic looking; the vesicle was incised and from the dark bloody fluid which discharged, a swab culture was made on Loeffler's medium, a tube of which I fortunately had in my valise; I also snipped out a small piece of the necrotic area, which after dressing the wound I took to my laboratory; the small piece of necrotic tissue was stirred about in melted agar, plated in Petri dishes and put in an incubator at 37° C. That afternoon the patient was much easier, temperature 101° F., pulse 110, urine contained five per cent. of albumin and a large number of small and large hyaline, epithelial and granular casts.

October 12th, agar plate showed a number of characteristic anthrax colonies; cover glass preparations were made from several of the colonies and from the growth in the culture tube; all showed a large, thick bacillus with squared ends, which stained easily, but no spores could be demonstrated in the bacilli. The diagnosis was now clear, my patient was suffering with anthrax (malignant carbuncle); I hurried to her home and found her suffering with increased pain, very nervous, temperature 102.2° F., pulse 108, and had slept very little during the night; wound showed necrotic tissue in all of the area occupied by the hemorrhagic vesicle, but no new vesicles had formed; wound was anesthetized with five per cent. cocaine solution and the entire necrotic area thoroughly curretted, then cauterized with pure carbolic acid, washed in sterile water and dressed with sublimate gauze.

Temperature returned to the normal on the third day; swelling of hand and arm soon disappeared, but the wound on the finger healed slowly.

Patient made an uninterrupted recovery.

The puzzling question in this case is, where did the infectious material come from? Was it conveyed to the wound by the splinter, the pin, the brush, the knife with which the wound was cleansed, or in some other way?

The drainage board from which the splinter came, is of slate with grooves cut in it which deepen as they approach the sink. Meat when it comes from the butcher is sometimes placed on the drainage board, and it is within the range of possibility that anthrax bacilli were sown into it from a piece of diseased meat. However, cultures made from scrapings of the board failed to show the anthrax bacillus. The pin was flamed before being used to dig out the splinter. The scrubbing-brush was a new one, having been purchased that morning, and its bristle were not of animal, but of some kind of vegetable fibre. The knife with which the wound was scraped was first dipped in alcohol, and the alcohol burned off before using, which insures sterilization.

Anthrax is very rare among cattle in this region. In 1895, however, quite a number of cases of the disease were discovered among the dairy cattle of the city, but it was quickly suppressed by the energetic action of Health Commissioner Dr. Max C. Starkloff, who quarantined all infected stables and destroyed and incinerated all diseased animals. To the knowledge of the health authorities, no case of the disease has occurred among cattle in this vicinity since that date. A case of the disease in man was reported from St. Louis county in June, 1900, by Drs. Elsworth Smith and H. G. Mudd. The origin of the infection in this case is unknown, which must also be declared of my case.

The bacteriologic examination of bacillus found in necrotic tissue from Mrs. Z., was as follows:

Bacillus, square ends, 3 to 5 m. in length. Isolated and in chain. Nonmotile. Forms spores in center of rod.

Stains easily with watery solutions of the basic aniline dyes. Decolorizes slowly when stained according to Gram.

Spores stain with Ziehls' carbolic fuchsin.

It grows freely on broth, forming a thick white granular deposit, with small flocculent masses clinging to side of tube.

Gelatine plate after six days at 20° C. shows small and large colonies, nearly all showing small amount of liquefaction; colonies under low power of microscope show finely, granular center with wavy threads extending out at some length from center, giving the so-called Medusa head appearance.

Gelatine cultures show a yellowish white growth along the thrust, with small spiculæ of growth extending out at right angles from it, giving a root-like appearance.

Glucose gelatine, slowly liquefied but no gas produced.

Milk, coagulated and casein liquified.

Potato, thick smeary yellowish brown growth.

Guinea pig weighing 465 grammes, injected subcutaneously into abdominal wall with 1 cc. of 48-hour old broth culture. Died in sixty hours.

On post-mortem made the same day, the abdomen was found to be swollen, the swelling extending to chest wall. Subcutaneously tissue, semi-fluid and gelatinous, with ecchymotic spots scattered through it. Lungs pale. Heart cavities distended with blood. Liver somewhat enlarged, with cloudy swelling. Spleen very much enlarged, dark, soft, friable. Kidneys congested.

Bacillus found in smear cover glass preparations made from heart blood and spleen pulp.

Diagnosis—Bacillus anthracis.

